# Associations of BMI and Body Fat with Urine Metabolome in Adolescents Are Sex-Specific: A Cross-Sectional Study

**DOI:** 10.3390/metabo10080330

**Published:** 2020-08-13

**Authors:** Christian Brachem, Julia Langenau, Leonie Weinhold, Matthias Schmid, Ute Nöthlings, Kolade Oluwagbemigun

**Affiliations:** 1 Unit of Nutritional Epidemiology, Department of Nutrition and Food Sciences, Rheinische Friedrich-Wilhelms-University Bonn, 53115 Bonn, Germany; langenau@uni-bonn.de (J.L.); noethlings@uni-bonn.de (U.N.); koluwagb@uni-bonn.de (K.O.); 2 Institute for Medical Biometry, Informatics and Epidemiology (IMBIE), University Hospital Bonn, 53127 Bonn, Germany; weinhold@imbie.meb.uni-bonn.de (L.W.); matthias.schmid@imbie.uni-bonn.de (M.S.)

**Keywords:** metabolomics, adolescents, body composition, sex-specific, body mass index, body fat

## Abstract

Epidemiologic studies examining the relationship between body composition and the urine metabolome may improve our understanding of the role of metabolic dysregulation in body composition-related health conditions. Previous studies, mostly in adult populations, have focused on a single measure of body composition, body mass index (BMI), and sex-specific associations are rarely explored. We investigate sex-specific associations of two measures of body composition—BMI and body fat (BF)—with the urine metabolome in adolescents. In 369 participants (age 16–18, 49% female) of the Dortmund Nutritional and Anthropometric Longitudinally Designed (DONALD) study, we examined sex-specific associations of these two measures of body composition, BMI and BF, and 1407 (467 unknown) 24 h urine metabolites analyzed by untargeted metabolomics cross-sectionally. Missing metabolites were imputed. We related metabolites (dependent variable) to BMI and BF (independent variable) separately using linear regression. The models were additionally adjusted for covariates. We found 10 metabolites associated with both BMI and BF. We additionally found 11 metabolites associated with only BF, and nine with only BMI. None of these associations was in females. We observed a strong sexual dimorphism in the relationship between body composition and the urine metabolome.

## 1. Introduction

Overweightness (including obesity) has reached epidemic proportions. Approximately 39% of the adult human population is overweight (BMI (body mass index) ≥ 25 to < 30 kg/m^2^) or obese (BMI ≥ 30 kg/m^2^) [1]. The global prevalence of overweightness (BMI > +1 standard deviation above the median) among adolescents aged 10 to 19 years has increased steadily over the last 40 years, from 4.3% in 1975 to 17.3% in 2016 [1,2]. Current research suggests that overweightness and obesity contribute to the increasing risk of chronic diseases [1]. The global burden of disease study estimated that in 2015, roughly 7% of deaths from any cause and roughly 5% of disability-adjusted life-years globally were due to high BMI [3]. Metabolic dysregulation, in addition to inflammation and insulin resistance, may mediate the link between overweightness and many chronic diseases, like Type 2 diabetes or cardiovascular diseases. There is mounting evidence that these links are already present in adolescents [1,4,5], implying an increase in risk of future incidence of chronic diseases. Therefore, it is important to find the metabolic changes already present in adolescence, and to understand the link between overweightness and disease progression in later life.

Being overweight is known to be related to metabolic changes—for example, through body fat functioning as an endocrine organ, producing adipokines like leptin or visfatin [6,7]. Additionally, past studies have shown that overweightness is likely to be a causal influence on the metabolome phenotype [8]. A recent review [7] that summarizes the current knowledge of the metabolomic signature of adult obesity concluded that many metabolite groups are altered, including sexual steroids, amino acids, and acylcarnitines, among others. Interestingly, only a few epidemiological studies have explored the relationship between body composition and the metabolome in adolescents [9,10]. Cho et al. [9] quantitatively measured the global metabolic repertoire in adolescents, and showed that endogenous metabolites and inflammation-related metabolites are related to body composition. Saner et al. [10] investigated metabolomic profiles in obese children and adolescents (ages 6 to 18), and found associations in post-pubertal males of several metabolites, including fatty acids, triglycerides, isoleucine, leucine, and glycoprotein with obesity measures. However, overall evidence is scarce calling for more studies profiling the adiposity metabolome, preferably by untargeted methods.

It is well-known that the body composition of adolescents is sex-specific [5]. While BMI tends to be comparable between males and females, body fat in females is physiologically higher starting in late puberty (Tanner stages IV and V). In addition, a sexual dimorphism in metabolism is well recognized [11]. Thus, investigating sex differences may reveal pathophysiologically relevant variations, with potential implications for overweightness- or obesity-related health conditions.

We decided to investigate two different measures of body composition to increase our confidence in the metabolite–body composition associations that are present for both measures. We used BMI, as it is the most widely used measure for body composition in observational studies [5,12]. It is well-understood that BMI is a good marker for body composition on the population level [13]. Specifically, in an adolescent population it has been demonstrated that BMI categories correctly identify children with excess body fat in roughly 85% of cases [5]. However, it has well-documented shortcomings regarding body fat distribution [14,15]. To address these shortcomings, we also used body fat percentage, as estimated with skinfold measurements.

Here, we explored the a priori, sex-stratified relationship between these two measures of body composition, BMI and BF, and the urine metabolome cross-sectionally among adolescents.

## 2. Results

### 2.1. Basic Characteristics

The basic characteristics of the participants (180 females, 189 males) are shown in Table 1. Females had a higher BF, were less physically active, consumed fewer calories, and were less likely to be overweight than males. Males were less likely to be current alcohol consumers, and more frequently their mothers were employed and of higher educational status. Roughly 20% of males and 13% of females were overweight (BMI ≥ 25).

### 2.2. Linear Regression Models

#### 2.2.1. Summarizing Metabolites into Groups Using Independent Component Analysis

We kept the first seven independent components (IC), according to the scree plot. The composition of the extracted components are recorded in Table A1. In our sample, no IC was associated with BMI or BF for either sex. A table of β-estimates with confidence limits can be found in Table A2.

#### 2.2.2. Metabolites Associated with Both BMI and BF

**Figure 1:**
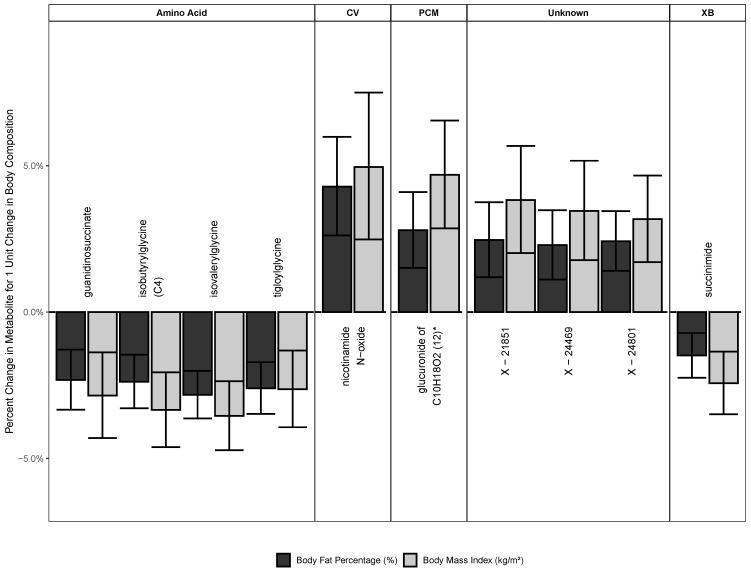
Metabolites associated with body mass index (BMI) and body fat (BF). Estimates are back-transformed linear regression beta coefficients, regressing metabolites on body composition (BMI or BF). BMI is measured in kg/m^2^ and body fat in percent. Abbreviations: CV, cofactor and vitamins; PCM: partially characterized molecules; XB: xenobiotics.

There were 10 metabolites (0.8% of metabolites analyzed) significantly associated with both BMI and BF in males, and zero metabolites in females (Figure 1). A table of β-estimates with confidence limits can be found in Table A3. The estimates presented here are back-transformed from the log-scale.

There were four amino acids associated significantly with BMI and BF: guanidinosuccinate (negative, BMI: 0.97 (0.96 to 0.99), BF: 0.98 (0.97 to 0.99)), isobutyrylglycine (C4) (negative, BMI: 0.97 (0.95 to 0.98), BF: 0.98 (0.97 to 0.99)), isovalerylglycine (negative, BMI: 0.96 (0.95 to 0.98), BF: 0.97 (0.96 to 0.98)), and tigloylglycine (negative, BMI: 0.97 (0.96 to 0.99), BF: 0.97 (0.97 to 0.98)).

In the super-pathway of cofactors and vitamins, nicotinamide N-oxide (positive, BMI: 1.05 (1.02 to 1.08), BF: 1.04 (1.03 to 1.06)) was associated. Additionally, the xenobiotic succinimide (negative, BMI: 0.98 (0.97 to 0.99), BF: 0.99 (0.98 to 0.99)) was associated with both BMI and BF significantly.

Furthermore, we found significant associations with both BMI and BF for the partially characterized molecule glucuronide of C_10_H_18_O_2_ (12) (positive, BMI: 1.05 (1.03 to 1.07), BF: 1.03 (1.02 to 1.04)) as well as the unknown metabolites X-21851 (positive, BMI: 1.04 (1.02 to 1.06), BF: 1.02 (1.01 to 1.04)), X-24469 (positive, BMI: 1.03 (1.02 to 1.05), BF: 1.02 (1.01 to 1.03)), and X-24801 (positive, BMI: 1.03 (1.02 to 1.05), BF: 1.02 (1.01 to 1.03)).

#### 2.2.3. Metabolites Associated with Either BMI or BF

**Figure 2:**
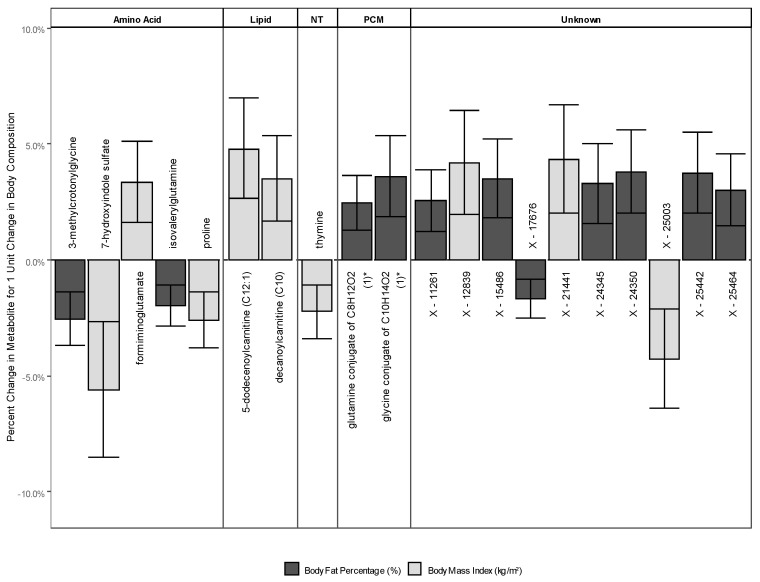
Metabolites associated with either BMI or BF. Estimates are back-transformed linear regression beta coefficients, regressing metabolites on body composition (BMI or BF). BMI units are kg/m^2^ and body fat units are percent. Abbreviations: NT, nucleotide; PCM, partially characterized molecules.

There were 20 metabolites (1.6% of metabolites analyzed) significantly associated with either BMI or BF. Of these, 11 were associated with BF and nine with BMI. All 20 associations were in males, none in females. A graphical representation of these results is presented in Figure 2. In Table A4, we present β-estimates and confidence intervals for all metabolites. The estimates presented here are back-transformed from the log-scale.

### 2.3. Metabolites Associated with BMI

The amino acids formiminoglutamate (positive, BMI: 1.03 (1.02 to 1.05)), 7-hydroxyindole sulfate (negative, BMI: 0.94 (0.92 to 0.97)), and proline (negative, BMI: 0.97 (0.96 to 0.99)) were associated with BMI. Additionally, the nucleobase thymine (BMI: 0.98 (0.97 to 0.99)) was associated negatively with BMI. Two lipids were significantly associated with BMI: decanoylcarnitine (C10) (positive, BMI: 1.04 (1.02 to 1.05)) and 5-dodecenoylcarnitine (C12:1) (positive, BMI: 1.05 (1.03 to 1.07)). Three unknown metabolites (X-12839 (positive, BMI: 1.04 (1.02 to 1.06)), X-21441 (positive, BMI: 1.04 (1.02 to 1.07)), and X-25003 (negative, BMI: 0.96 (0.94 to 0.98))) were associated with BMI.

### 2.4. Metabolites Associated with BF

The amino acids 3-methylcrotonylglycine (negative, BF: 0.97 (0.96 to 0.99)) and isovalerylglutamine (negative, BF: 0.98 (0.97 to 0.99)) were significantly associated with BF. The energy metabolite malate (negative, BF: 0.97 (0.96 to 0.99)) was significantly associated with BF as well. Additionally, there were two partially characterized molecules (glutamine conjugate of C_8_H_12_O_2_ (1) (positive, BF: 1.02 (1.01 to 1.04)) and glycine conjugate of C_10_H_14_O_2_ (1) (positive, BF: 1.04 (1.02 to 1.05)) and seven unknown metabolites (X-11261 (positive, BF: 1.03 (1.01 to 1.04)), X-15486 (positive, BF: 1.04 (1.02 to 1.05)), X-17676 (negative, BF: 0.98 (0.97 to 0.99)), X-24345 (positive, BF: 1.03 (1.02 to 1.05)), X-24350 (positive, BF: 1.04 (1.02 to 1.06)), X-25442 (positive, BF: 1.04 (1.02 to 1.06)), and X-25464 (positive, BF: 1.03 (1.01 to 1.05))) that were significantly associated.

## 3. Discussion

The current study explores the sex-specific cross-sectional associations of two measures of body composition, BMI and BF, and the urine metabolome and urine metabolite patterns (ICs) in adolescent boys and girls. Approximately 2.4% of the urine metabolome was associated with body composition in boys; no association was seen in girls. Our results underscore the presence of changes in the urine metabolome in relation to body composition already in adolescence. To our knowledge, this is the first study to relate two measures of body composition to the urine metabolome in adolescents. Our results strongly suggests sex-specificity in associations.

We advise the reader that the results of the present study were exploratory, and therefore should not be overemphasized. Any interpretation we give here in relation to the biological process may only be seen as one of many possible explanations for the reported associations. In fact, many of the reported compounds have not been reported in conjunction with body composition before. A more in-depth investigation of these single compounds is, however, outside of the scope of this study. We found 10 metabolites that related to both measures of body composition in males, and none in females. These metabolites were guanidinosuccinate, isobutyrylglycine (C4), isovalerylglycine, tigloylglycine, nicotinamide N-oxide, glucuronide of C_10_H_18_O_2_ (12), X-21851, X-24469, X-24801, and succinimide. Nicotinamide N-oxide [1] and tigloylglycine [2] have been associated with BMI in prior studies. The other eight molecules are reported in association with body composition here for the first time. Additionally, we found 20 metabolites associated with either BMI or BF. When metabolites are significantly associated with both measures of body composition, we should have higher confidence in their association. As both measures have their own unrelated measurement error while measuring different aspects of the same concept (body composition), a significant association with both BMI and BF should indicate that it is more likely related to this underlying concept. The metabolites associated with only BMI or BF, however, were all associated in the same direction with the other body composition measurement. Additional discussion of these metabolites can be found in Table A5.

In general, our results reinforced the idea of sexual dimorphism in metabolism. The stronger association in males is consistent with previous studies in mice [3], adults [4,5], and adolescents [6], as well as our own recent findings within this study population [7]. One potential explanation is that sex hormones might modify the relationship between body composition and the urine metabolome. Specifically, prior studies have shown changes in the type of body composition and overall obesity in relation to sex hormones and displaying sexual dimorphism in their mode of effect [8,9,10,11]. Furthermore, the sexual dimorphism in the urine metabolome is well-documented [4,12,13,14,15]. As sex hormones play an important role in many metabolic pathways, e.g., they have been shown to regulate the liver energy homeostasis [16], an interaction between sex hormones, body composition, and the urine metabolome is plausible. Another explanation, as was shown for urine cortisol levels [5], is that sex differences relate to other factors of metabolism, such as enzyme activity. Wang et al. [17] showed that lipid and lipoprotein metabolism is in fact independent of sex hormone administration, even though there are significant sex differences; however, the mechanism remains to be elucidated. The specific mechanism of sex difference in metabolism might therefore differ for different pathways, and deserves to be studied further. Our results may help to explain sex differences in weight-related health conditions.

We used independent component analysis (ICA) to summarize metabolites into fewer components in the current analysis. We chose ICA because the components are statistically independent, and their interpretation in biological processes allows for the mixture of different pathways and processes that contribute to the living system. Because metabolomics takes a snapshot of these processes and systems, these components hold a large value for understating of processes. In the current study, none of the ICs we retained were associated with body composition. This suggests that body composition influences specific metabolic pathways, and not a mixture of different pathways captured by the ICA.

Guanidinosuccinate is produced by the oxidation of argininosuccinic acid, and was associated with higher measures of body composition in males. The oxidation of guanidinosuccinate occurs favorably with increased levels of urea, and results in a decline of hepatic levels of arginine [18]. It is well-known that the urea cycle is dysregulated with higher adiposity [19]; therefore, reduced renal function compared to the average adolescent may partly explain our findings. Guanidinosuccinate may be a marker of the kidneys’ ability to eliminate urea, particularly in males.

Isobutyrylglycine (C4) is a short-chain acylglycine in the catabolism of leucine, isoleucine, and valine. In newborn screenings, elevated levels of this metabolite are used to diagnose isobutyryl-CoA dehydrogenase deficiency [20]. Since isobutrylglycine levels decrease with higher BMI and BF, isobutyryl-CoA dehydrogenase might be upregulated with elevated measures of body composition. Alternatively, smaller amounts of leucine, isoleucine, and valine might be catabolized in individuals with abnormal adiposity. However, the present association was independent of these metabolites.

Isovalerylglycine is an acyl glycine that is produced in the catabolism of leucine [18]. Higher BMI and BF are associated with the metabolism of leucine in rats [21]; however, no study to date exists in humans. This metabolite has also been suggested as a biomarker for cheese consumption [22]. Although we did not specifically adjust for cheese intake, the fact that we adjusted for macronutrient intake suggests that our finding is independent of cheese intake.

Tigloylglycine is an acylglycine that is an intermediate of the isoleucine catabolism [18]. Like isovalerylglycine, it was suggested as a biomarker for the consumption of cheese [18]. Again, we adjusted for nutrition, so an association because of cheese consumption is unlikely. Urinary acylglycine decreases with higher BMI have been documented before [2]. Similar to other leucine, isoleucine, and valine metabolites, the enzyme metabolizing this compound might be upregulated, or the overarching pathway of branched-chain amino acid (BCAA) catabolism might be dysregulated.

BCAAs have a well-documented association with higher markers of body composition: increased blood levels of BCAAs correlated with higher levels of body composition [19]. A recent study by Elliot et al. [23] reported associations between increased urine levels of leucine, isoleucine, and valine and BMI. Additionally, they reported lower levels of ketoleucine with higher BMI. Ketoleucine is the first metabolic product in the energy use of leucine [24]. The metabolites we found that decreased with higher measures of body composition are downstream metabolites of BCAAs, which are produced through similar processes as ketoleucine from leucine, namely when their respective BCAA is used for energy in skeletal muscle. As BCAAs are not the first energy source muscles use in response to physical activity, increased blood levels of BCAAs and decreased levels of their energy pathway downstream products are in line with decreased physical activity and overabundance of other energy sources in persons with higher measures of body composition.

Nicotinamide N-oxide is a precursor of nicotinamide adenine dinucleotide (NAD) and a catabolite of nicotinamide [18,25]. Increased urine nicotinamide N-oxide is associated with high-fat, diet-induced obesity in mice [26]. In humans, serum levels of another nicotinamide was positively associated with BMI and waist circumference [1]. This finding suggests that in individuals with higher measures of body composition, there is a nicotinamide overload, or enzymes catabolizing nicotinamide to nicotinamide N-oxide are overexpressed or hyper-activated. However, our result is independent of nicotinamide, which favors the latter explanation.

Succimide is commonly found in anticonvulsant drugs [18]. The fact that a common side effect of anticonvulsant drugs are changes in weight [27] might provide a potential explanation for the association with adiposity.

Additionally, there are no available data on the relationship between the unknown metabolites X-21851, X-24469, and X-24801, or the partially characterized metabolite glucuronide of C10H18O2 (12) and body composition. Besides, since they are without biochemical identities, or only partially characterized, it is difficult to provide explanations. Nevertheless, with the rapidly developing field of metabolomics, the identification of these metabolites should not be far from sight.

The present study has some notable strengths. We investigated the associations between body composition and the urine metabolome using two measures of body composition, in order to achieve a more comprehensive relationship between body composition and alteration in urine metabolites. The sex-specific investigation defined a priori also ensures that sex-specific relationships are well explored. Additionally, we used 24 h urine samples in a comparatively large study population to study the urine metabolome with an untargeted approach. To limit the possibility of false positives that untargeted approaches entail, we controlled for multiple testing by holding the False Discovery Rate (FDR) at 5%.

However, we acknowledge several limitations to the study. First, our participants are all Caucasians (Germans), residing in a large city (Dortmund) and surroundings, mostly from a high socioeconomic background. Thus, the generalizability of our findings is limited. Further, our study sample had very few individuals in the extremes of body composition, namely in the underweight (BMI < 18.5) and the obese (BMI ≥ 30) classifications, our findings may only be generalizable to individuals with normal and overweight body composition status. More associations of metabolites with BF as compared to BMI may also be due to BF having a larger variation in our study sample. Additionally, we cannot rule out residual confounding by either unknown or unmeasured (for example, genetic influences) factors. Lastly, because we only had one measurement of the urine metabolome, we were not able to establish a relationship of body composition and variability in the urine metabolome.

Future research should try to identify the unknown or partially characterized molecules that were associated in this study, as they have potential to help elucidate the biological mechanisms of the relationship between body composition and metabolic function on the pathway to health outcomes. Additionally, more studies are needed that stratify their metabolomic analysis by sex, in order to increase our understanding of the physiological differences in metabolism between males and females. Furthermore, future studies should try to replicate our findings in an independent adolescent population, and try to extend the analysis to a longitudinal design to elucidate the temporal relation of body composition with urine metabolome. Additionally, it would be interesting to evaluate differences between the blood and urine metabolome in a similar study setting, preferably in the same participants. Overall, metabolomics would benefit greatly from more unified data analysis approaches to facilitate meta-analysis of different cohorts. Lastly, a similar analysis carried out in a cohort with a larger proportion of overweight and obese participants would help to disentangle the gradient relationship between body composition and the urine metabolome.

## 4. Materials and Methods

### 4.1. Study Design

The present analysis is conducted within the Dortmund Nutritional and Anthropometric Longitudinally Designed (DONALD) study. Briefly, the DONALD study is a longitudinal open cohort study with the aim of analyzing detailed data on diet, growth, development, and metabolism from infancy to adulthood [28]. All study participants were invited to the study center on a regular basis, every 3 months until their first birthday, biennially in their second year, and annually thereafter. The anthropometric measurements are conducted by experienced nurses [28]. Data collected includes demographic, family, and socioeconomic characteristics, as well as anthropometric measurements, such as height, body weight, skin fold thickness, and 3 day weighed dietary records [28]. Informed written consent was obtained from parents and from participants themselves on reaching adolescence. The ethics committee of the University of Bonn, Germany (project identification: 098/06) approved the study.

### 4.2. Study Participants

The current study sample were DONALD participants from a previous study that explored BMI trajectories [29]. These 689 individuals are singletons, full-term (37 to 42 weeks of gestation), and had a birth weight of at least 2500 g. Of these, 369 participants had a 24 h urine samples between the ages of 16 to 18, from which the urine metabolome were profiled by an untargeted metabolomics approach.

### 4.3. Variable Assessment

#### 4.3.1. Outcome: Untargeted Metabolomic Profiling of the Urine Metabolome

Ultra-high-performance liquid chromatography–tandem mass spectroscopy (UPLC-MS/MS) was used to identify metabolites in the 24 h urine samples. Peak identification was done in the propriety Laboratory Information Management System (LIMS) of Metabolon Inc. (Morrisville, NC, USA). Compounds were identified by comparison of their retention time/index (RI), mass-to-charge ratio (*m*/*z*), and chromatographic data (e.g., MS/MS spectral data) to library standards. Structurally unknown biochemicals were identified by occurrence. Peaks were quantified using area-under-the-curve and normalized with block correction corrected for inter-day instrument tuning differences. Further details on the methodology of the metabolic profiling have been reported elsewhere [30]. This analysis resulted in 1407 annotated features used in this analysis.

#### 4.3.2. Exposure: Body Composition Measures

Body composition parameters were examined at every follow-up by experienced nursing staff. BMI was calculated using height and weight. BF was calculated from four skin-fold thickness measurements (biceps, triceps, iliaca, and scapula), using age, puberty status, and sex-specific equations from Deurenberg et al. [31].

#### 4.3.3. Covariates

We constructed a directed acyclic graph (DAG; cf. supplement) to assess the minimally sufficient sets of variables to use for covariate adjustment in the analysis of the present data.

Family and socioeconomic characteristics around birth were assessed at the first study visit in the DONALD study, at around three months after birth. We included maternal employment (full- or part-time employment vs. no employment), maternal education (>12 vs. ≤12 years of education), smoking in the household (yes vs. no), maternal BMI (kg/m^2^), and duration of breast-feeding (weeks). Dietary intake was assessed annually by three-day weighted dietary records. We calculated individual means of daily calorie and macronutrient intake, using our continuously updated in-house food composition database LEBTAB [32]. Macronutrient intake was estimated as percent of calories consumed. Using the method described by Schofield [33], the metabolic equivalents of task-hours (met-h) were determined from basal rates. The expanded met-h per week were subsequently calculated from the Adolescent Physical Activity Recall Questionnaire (APARQ) [34]. Alcohol and smoking status were assessed via questionnaire. Participants were grouped into current, former, and never drinkers (or smokers). Missing values were filled backwards for never drinkers (or smokers) and forwards for current and former drinkers (or smokers). Backwards filling means “never” consumption was used later to fill in missing values at time points prior to the non-missing answer, e.g., “never” consumption at age 20 was used to fill a missing value at age 16, since the participant was never a consumer. Forward filling refers to the same concept but forwards in time, e.g., current alcohol consumption at age 15 was used to fill a missing value for the current alcohol consumer at age 17.

### 4.4. Statistical Analysis

Statistical Analysis was performed using SAS software (Version 9.4 of the SAS System for Windows, copyright 2002–2012 SAS Institute Inc., Cary, NC, USA) and R software (Version 3.6) [35]. All analyses were a priori and were stratified by sex.

#### 4.4.1. Data Pre-Treatment

Metabolite concentration were normalized by urine osmolality and rescaled to set the median equal to 1. Because the distributions of most metabolites were skewed, we performed natural log-transformation. We excluded metabolites with more than 20% of missing values (*n* = 140) to keep the data quality acceptable. Missing values for the remaining 1267 metabolites were imputed.

#### 4.4.2. Imputation of Missing Values

Missing values for all variables were imputed with multiple imputations (as implemented in the R package “mice” [36]) with 10 imputations and five iterations. We used the random forest method built into the “mice” package. The variables used as predictors for imputation for each “to be imputed” variable were selected according to the suggestions of the authors of the mice package [37]: variables were selected as predictors in the imputation if they had at least a correlation of *r* = 0.35 with the “to be imputed” variable, and at least 70% of the observations used for imputation had complete data. Imputation was stratified by sex.

#### 4.4.3. Summarizing Metabolites into Groups Using the Independent Component Analysis

To reduce intercorrelation among metabolites, we summarized them into fewer interpretable components using independent component analysis (ICA). We used the “icafast” function from the “ica” Package [38] to perform the ICA, stratified by sex. We used the mean across all imputed datasets to extract the component model. The number of components to include in the regression analysis was selected by visual inspection of the scree plot. We then calculated the component scores for each imputed dataset according to the mean model. We characterized the components by their correlation with the metabolites, using the top 20 most correlated metabolites.

#### 4.4.4. Linear Regression Model

In order to model the associations between BMI and BF and the urine metabolome, we fitted a linear regression model for each of the 1177 log-transformed metabolites and seven ICs as dependent variables, and either BMI or BF as independent variables. The models were additionally adjusted for the minimally sufficient set suggested by the DAG, which are physical activity (met-h/week), age, alcohol and smoking status, nutrition (total energy (kcal), protein (%kcal), fat (%kcal), and carbohydrates (%kcal)), smoking household, maternal occupation at study entry, maternal education, breastfeeding duration, and maternal BMI at study entry. If not otherwise specified, variables were measured during the same follow-up as the 24 h urine sample was taken. We performed multiple testing corrections by controlling the false discovery rate at five percent with the Benjamini–Hochberg procedure [39]. Metabolites associated with both BMI and BF were considered a signature of body composition.

## 5. Conclusions

In conclusion, 10 metabolites (10 in males, none in females) were associated with both measures of body composition, which could collectively be considered a metabolic signature of body composition. The sexual dimorphism in the relationship between body composition and the urine metabolome may explain sex differences in body composition-related health conditions.

## Figures and Tables

**Table 1:** Characteristics of 369 Dortmund Nutritional and Anthropometric Longitudinally Designed (DONALD) study participants aged 16 to 18 years.

Variable	*n*	Total	Male	Female
		*n* = 369	*n* = 189	*n* = 180
Age (years)	369	17.3 (1.0)	17.2 (1.0)	17.4 (1.0)
Body Fat Percent	368	21.8 (8.0)	16.6 (5.6)	27.3 (6.4)
BMI (kg/m^2^)	369	22.2 (3.7)	22.5 (4.0)	21.9 (3.2)
Overweight (BMI ≥ 25): Yes	369	62 (16.8%)	39 (20.6%)	23 (12.8%)
Metabolic Equivalent of Task-Hours (met-h/week)	207	41.3 (37.0)	45.9 (43.3)	36.6 (28.8)
Calories (kcal)	364	2189.2 (616.7)	2545.8 (565.4)	1816.5 (415.4)
Protein (%kcal)	364	13.9 (2.8)	14.1 (2.7)	13.7 (2.8)
Fat (%kcal)	364	33.7 (6.6)	33.9 (7.0)	33.6 (6.1)
Carbohydrates (%kcal)	364	50.5 (6.9)	49.8 (7.4)	51.2 (6.3)
Smoking Status	118			
Never		56 (15.2%)	28 (14.8%)	28 (15.6%)
Former		35 (9.5%)	13 (6.9%)	22 (12.2%)
Current		27 (7.3%)	12 (6.3%)	15 (8.3%)
Alcohol Status	155			
Never		8 (2.2%)	5 (2.6%)	3 (1.7%)
Former		11 (3%)	5 (2.6%)	6 (3.3%)
Current		136 (36.9%)	65 (34.4%)	71 (39.4%)
Maternal Occupation: Working (full or part-time)	364	222 (60.2%)	122 (64.6%)	100 (55.6%)
Maternal Education: >12 Years of Education	365	190 (51.5%)	101 (53.4%)	89 (49.4%)
Breastfeeding Duration (weeks)	363	25.0 (18.3)	24.3 (18.9)	25.7 (17.7)
Maternal Gestational Weight Gain (kg)	348	12.8 (4.1)	12.7 (4.2)	13.0 (4.1)
Maternal BMI (kg/m^2^) (kg/m^2^)	358	23.7 (3.7)	23.8 (3.5)	23.7 (3.9)
Smoking Household: Yes	265	86 (23.3%)	43 (22.8%)	43 (23.9%)

Data are presented as mean (standard deviation) for continuous measures and *n* (column percent) for categorical measures. Available *n* values differ because of missing data.

## Appendix A: Additional Information on Independent Components

**Table A1:** Names of the Independent Components.

IC	Constructed by
Sex: female	
IC1	Amino acid (10), unknown (5), lipid (2), nucleotide (2), and xenobiotics (1)
IC2	Amino acid (6), xenobiotics (5), unknown (5), partially characterized molecules (3), and peptide (1)
IC3	Unknown (10), xenobiotics (4), amino acid (3), peptide (2), and lipid (1)
IC4	Unknown (9), xenobiotics (5), amino acid (3), and lipid (3)
IC5	Amino acid (6), lipid (5), unknown (5), partially characterized molecules (2), nucleotide (1), and xenobiotics (1)
IC6	Unknown (12), amino acid (3), xenobiotics (2), lipid (1), partially characterized molecules (1), and peptide (1)
IC7	Amino acid (5), xenobiotics (5), unknown (5), lipid (2), nucleotide (1), partially characterized molecules (1), and peptide (1)
Sex: male	
IC1	Unknown (9), xenobiotics (4), amino acid (3), nucleotide (2), energy (1), and lipid (1)
IC2	Amino acid (5), lipid (5), nucleotide (3), xenobiotics (2), unknown (2), carbohydrate (1), partially characterized molecules (1), and peptide (1)
IC3	Unknown (8), amino acid (5), xenobiotics (3), lipid (2), carbohydrate (1), and energy (1)
IC4	Xenobiotics (13), unknown (6), and lipid (1)
IC5	Xenobiotics (7), unknown (6), partially characterized molecules (4), amino acid (1), lipid (1), and nucleotide (1)
IC6	Unknown (10), lipid (4), partially characterized molecules (4), amino acid (1), and xenobiotics (1)
IC7	Xenobiotics (8), unknown (6), carbohydrate (4), lipid (1), and partially characterized molecules (1)

**Table A2:** Regression Coefficients for the ICs and body composition.

IC	β Body Mass Index	95% CI	*p* (FDR)	β Body Fat Percent	95% CI	*p* (FDR)
Sex: Female						
IC1	1.025	0.890 to 1.180	0.991	0.996	0.914 to 1.086	0.991
IC2	0.974	0.832 to 1.141	0.991	1.030	0.943 to 1.125	0.991
IC3	0.979	0.838 to 1.143	0.991	1.024	0.946 to 1.109	0.991
IC4	0.945	0.829 to 1.076	0.991	1.014	0.942 to 1.093	0.991
IC5	0.885	0.733 to 1.070	0.991	1.054	0.942 to 1.179	0.991
IC6	0.930	0.814 to 1.064	0.991	1.057	0.979 to 1.141	0.991
IC7	0.928	0.799 to 1.078	0.991	1.034	0.949 to 1.126	0.991
Sex: Male						
IC1	1.012	0.903 to 1.135	1.000	0.986	0.893 to 1.088	1.000
IC2	0.969	0.880 to 1.067	1.000	1.001	0.923 to 1.086	1.000
IC3	1.045	0.940 to 1.162	1.000	0.974	0.891 to 1.064	1.000
IC4	0.980	0.877 to 1.095	1.000	1.053	0.958 to 1.157	1.000
IC5	0.993	0.896 to 1.100	1.000	1.039	0.959 to 1.127	1.000
IC6	0.956	0.872 to 1.048	1.000	1.015	0.943 to 1.093	1.000
IC7	0.991	0.889 to 1.104	1.000	0.952	0.870 to 1.041	1.000

Estimates were generated from linear regression models, with independent components as the dependent variable and body mass index or body fat percent as the dependent variable. Multiple testing adjustments were performed by controlling the false discovery rate at 5%. Estimates are bac-transformed. The models were additionally adjusted for age, calories consumed, protein consumed, carbohydrates consumed, fat consumed, maternal gestational weight gain, maternal BMI, breastfeeding duration, smoking status, smoking household, alcohol status, maternal occupation, maternal education, metabolic equivalent of task-hours. Abbreviations: IC, independent component; FDR, False Discovery Rate

## Appendix B: Regression Coefficient Tables

**Table A3:** Metabolites associated with both body composition measures.

Biochemical	Sub Pathway	β Body Mass Index	95% CI	*p* (FDR)	β Body Fat Percent	95% CI	*p* (FDR)
Super-pathway: Amino Acid							
guanidinosuccinate	Guanidino and acetamido metabolism	0.971	0.957 to 0.986	0.046	0.977	0.967 to 0.987	0.014
isobutyrylglycine (C4)	Leucine, isoleucine, and valine metabolism	0.967	0.954 to 0.979	0.002	0.976	0.967 to 0.985	0.001
isovalerylglycine	Leucine, isoleucine, and valine metabolism	0.965	0.953 to 0.976	0.000	0.972	0.964 to 0.980	0.000
tigloylglycine	Leucine, isoleucine, and valine metabolism	0.974	0.961 to 0.987	0.036	0.974	0.965 to 0.983	0.000
Super-pathway: CV							
Nicotinamide N-oxide	Nicotinate and nicotinamide metabolism	1.050	1.025 to 1.075	0.030	1.043	1.026 to 1.060	0.001
Super-pathway: PCM							
Glucuronide of C10H18O2 (12) *	Partially characterized molecules	1.047	1.029 to 1.066	0.001	1.028	1.015 to 1.041	0.016
Super-pathway: Unknown							
X-21851		1.038	1.020 to 1.057	0.021	1.025	1.012 to 1.038	0.044
X-24469		1.035	1.018 to 1.052	0.025	1.023	1.011 to 1.035	0.044
X-24801		1.032	1.017 to 1.047	0.016	1.024	1.014 to 1.034	0.004
Super-pathway: XB							
Succinimide	Chemical	0.976	0.965 to 0.986	0.011	0.985	0.978 to 0.993	0.046

Estimates were generated from linear regression models with natural log-transformed biochemicals as the dependent variables, and body mass index or body fat percent as the dependent variable. Multiple testing adjustment was performed by controlling the false discovery rate at 5%. Estimates are back-transformed. The models were additionally adjusted for age, calories consumed, protein consumed, carbohydrates consumed, fat consumed, maternal gestational weight gain, maternal BMI, breastfeeding duration, smoking status, smoking household, alcohol status, maternal occupation, maternal education, metabolic equivalent of task-hours. * Compound was not identified by a standard, but we are confident in its identity. Abbreviations: CV, cofactors and vitamins; PCM, partially characterized molecules; XB, xenobiotics; FDR, False Discovery Rate.

**Table A4:** Metabolites associated with either BMI or BF.

Biochemical	Sub-Pathway	Body Mass Index	95% CI	*p* (FDR)	Body Fat Percent	95% CI	*p* (FDR)
**Super-Pathway: Amino Acid**							
Formiminoglutamate	Histidine metabolism	**1.033**	**1.016 to 1.051**	**0.041**	1.019	1.007 to 1.031	0.162
3-methylcrotonylglycine	Leucine, isoleucine, and valine metabolism	0.971	0.954 to 0.988	0.091	**0.975**	**0.963 to 0.986**	**0.021**
Isovalerylglutamine	Leucine, isoleucine, and valine metabolism	0.978	0.966 to 0.991	0.120	**0.980**	**0.972 to 0.989**	**0.016**
7-hydroxyindole sulfate	Tryptophan metabolism	**0.944**	**0.915 to 0.973**	**0.050**	0.961	0.940 to 0.982	0.060
proline	Urea cycle; arginine and proline metabolism	**0.974**	**0.962 to 0.986**	**0.023**	0.988	0.979 to 0.997	0.281
**Super-Pathway: Lipid**							
decanoylcarnitine (C10)	Fatty acid metabolism (acyl carnitine, medium chain)	**1.035**	**1.017 to 1.054**	**0.046**	1.023	1.010 to 1.036	0.083
5-dodecenoylcarnitine (C12:1)	Fatty acid metabolism (acyl carnitine, monounsaturated)	**1.048**	**1.027 to 1.070**	**0.009**	1.025	1.010 to 1.041	0.110
**Super-Pathway: Nucleotide**							
Thymine	Pyrimidine metabolism, thymine containing	**0.978**	**0.966 to 0.989**	**0.046**	0.987	0.979 to 0.995	0.169
**Super-Pathway: PCM**							
Glutamine conjugate of C8H12O2 (1) *	Partially characterized molecules	1.030	1.013 to 1.047	0.065	**1.025**	**1.013 to 1.036**	**0.021**
Glycine conjugate of C10H14O2 (1) *	Partially characterized molecules	1.044	1.019 to 1.069	0.071	**1.036**	**1.019 to 1.054**	**0.023**
**Super-Pathway: Unknown**							
X-11261		1.032	1.013 to 1.051	0.111	**1.025**	**1.012 to 1.039**	**0.045**
X-12839		**1.042**	**1.020 to 1.065**	**0.048**	1.024	1.009 to 1.040	0.175
X-15486		1.039	1.015 to 1.064	0.118	**1.035**	**1.018 to 1.052**	**0.021**
X-17676		0.981	0.969 to 0.993	0.142	**0.983**	**0.975 to 0.991**	**0.034**
X-21441		**1.043**	**1.020 to 1.067**	**0.047**	1.029	1.013 to 1.046	0.078
X-24345		1.040	1.015 to 1.065	0.123	**1.033**	**1.016 to 1.050**	**0.044**
X-24350		1.040	1.014 to 1.067	0.156	**1.038**	**1.020 to 1.056**	**0.020**
X-25003		**0.957**	**0.936 to 0.979**	**0.044**	0.976	0.960 to 0.992	0.192
X-25442		1.041	1.016 to 1.067	0.120	**1.038**	**1.020 to 1.055**	**0.015**
X-25464		1.039	1.017 to 1.062	0.076	**1.030**	**1.015 to 1.046**	**0.037**

Estimates were generated from linear regression models, with natural log-transformed biochemicals as the dependent variable and body mass index or body fat percent as the dependent variable. Multiple testing adjustments were performed by controlling the false discovery rate at 5%. Estimates are back-transformed. The models were additionally adjusted for age, calories consumed, protein consumed, carbohydrates consumed, fat consumed, maternal gestational weight gain, maternal BMI, breastfeeding duration, smoking status, smoking household, alcohol status, maternal occupation, maternal education, metabolic equivalent of task-hours. * Compund was not identified by a standard, but we are confident in its identity. Abbreviations: CV, cofactors and vitamins; PCM, partially characterized molecules; XB, xenobiotics. Associations in females are highlighted in italic. The significant association for each metabolite is highlighted in bold.

## Appendix C: Additional Discussion for Metabolites Associated with Either BMI or BF

**Table A5:** Additional discussion for metabolites associated with either BMI or BF.

Biochemical	Sub-Pathway	Sex	Body Composition	Discussion
**Super-Pathway: Amino Acid**				
7-hydroxyindole sulfate	Tryptophan metabolism	male	BMI	Part of the serotonin-related pathway of tryptophan [18]. A relationship to mood and depression, which has been documented to be influenced by weight and the perception thereof in adolescents [40], is a possible explanation for this association.
Formiminoglutamate	Histidine metabolism	male	BMI	Measurements in urine after oral application of histidine are used to determine folate deficiency [18]. Higher levels of this metabolite in the urine of individuals with higher adiposity might point to an increased need for folate. In fact, overweightness was previously shown to be associated with decreased levels of folate [41].
Proline	Urea cycle; arginine and proline metabolism	male	BMI	Proline was inversely associated with adiposity in our study. This is in agreement with findings in children, in which lower levels of the metabolite have been observed in overweight children [42], but is in contrast to findings in adults [43,44]. This suggests that the relationship of adiposity with proline varies with the developmental stage of life.
3-methylcrotonylglycine	Leucine, isoleucine, and valine metabolism	male	BF	A catabolite of leucine. Elevated levels of this metabolite in urine are usually found in patients with a deficiency of 3-methylcrotonyl-CoA carboxylase, an inborn error of the metabolism [18]. Decreased levels in our sample could be explained by hyperactivation of 3-methylcrotonyl-CoA carboxylase or disruption of the leucine metabolism.
Isovalerylglutamine	Leucine, isoleucine and valine metabolism	male	BF	No information
**Super Pathway: Lipid**				
5-dodecenoylcarnitine (C12:1)	Fatty acid metabolism (acyl carnitine, monounsaturated)	male	BMI	Medium-chain acylcarnitines (MCACs), see decanoylcarnitine (C10)
Decanoylcarnitine (C10)	Fatty acid metabolism (acyl carnitine, medium chain)	male	BMI	Decanoylcarnitine (C10) is a medium-chain fatty acid acylcarnitine that was significantly associated with higher measures of body composition. In fact, urine decanoylcarnitine has been shown to differentiate young men with normal weight from those with obesity [45], and differentiates individuals with metabolically healthy obesity from those with metabolically abnormal obesity [46]. Additionally, it is among a group of acylcarnitines that is positively related to fat oxidation [47]. It was suggested previously that high levels of medium-chain acylcarnitines (MCACs) reflect distal β- oxidation for energy use. C6 and C10 in particular are used as markers for MCAC flux [48]. Higher levels of MCAC have also been related to a disrupted branched-chain amino acid (BCAA) metabolism [49,50]. Additionally, increased levels of MCAC were suggested as markers for insulin resistance in overweight and obese individuals [51]. Increased levels of C10 in our sample are in line with the findings of previous studies in adults and children, reporting either higher levels of closely related acylcarnitines or C10 exactly [19]. However, most of these were using different tissues (e.g., blood or muscle fiber) as their biospeciminen [19].
**Super Pathway: Nucleotide**				
Thymine	Pyrimidine metabolism, thymine containing	male	BMI	Change within increasing adiposity is in line with cytosine per thymine change present in a single-nucleotide polymorphism (SNP) that is associated with BMI and BF [52]. This supports evidence that adiposity has a genetic component. Future studies should explore the relation between this SNP and adiposity
**Super-Pathway: PCM**				
Glutamine conjugate of C8H12O2 (1) *	Partially characterized molecules	male	BF	No information
Glycine conjugate of C10H14O2 (1) *	Partially characterized molecules	male	BF	No information
**Super-Pathway: Unknown**				
X-12839		male	BMI	No information
X-21441		male	BMI	No information
X-25003		male	BMI	No information
X-11261		male	BF	No information
X-15486		male	BF	No information
X-17676		male	BF	No information
X-24345		male	BF	No information
X-24350		male	BF	No information
X-25442		male	BF	No information
X-25464		male	BF	No information

* Compound was not identified by a standard, but we are confident in its identity. Abbreviations: BF, body fat percentage; BMI, body mass index; CV, cofactors and vitamins; PCM, partially characterized molecules; XB, xenobiotics.
